# Formation of Combustible Hydrocarbons and H_2_ during Photocatalytic Decomposition of Various Organic Compounds under Aerated and Deaerated Conditions

**DOI:** 10.3390/molecules191219633

**Published:** 2014-11-26

**Authors:** Sylwia Mozia, Aleksandra Kułagowska, Antoni W. Morawski

**Affiliations:** Institute of Chemical and Environment Engineering, West Pomeranian University of Technology, ul. Pułaskiego 10, 70–322 Szczecin, Poland; E-Mails: aheciak@zut.edu.pl (A.K.); amor@zut.edu.pl (A.W.M.)

**Keywords:** photocatalysis, hydrocarbons, hydrogen, organic substrates, Fe/TiO_2_

## Abstract

A possibility of photocatalytic production of useful aliphatic hydrocarbons and H_2_ from various organic compounds, including acetic acid, methanol, ethanol and glucose, over Fe-modified TiO_2_ is discussed. In particular, the influence of the reaction atmosphere (N_2_, air) was investigated. Different gases were identified in the headspace volume of the reactor depending on the substrate. In general, the evolution of the gases was more effective in air compared to a N_2_ atmosphere. In the presence of air, the gaseous phase contained CO_2_, CH_4_ and H_2_, regardless of the substrate used. Moreover, formation of C_2_H_6_ and C_3_H_8_ in the case of acetic acid and C_2_H_6_ in the case of ethanol was observed. In case of acetic acid and methanol an increase in H_2_ evolution under aerated conditions was observed. It was concluded that the photocatalytic decomposition of organic compounds with simultaneous generation of combustible hydrocarbons and hydrogen could be a promising method of “green energy” production.

## 1. Introduction

Over the past thirty years increased concerns over emissions of greenhouse gases and the depletion of non-renewable resources of fossil fuels has caused the necessity to look for new methods of energy production. From both the ecological and economical point of view conversion of waste and wastewaters into energy is especially desirable. One of the most promising and popular approaches is biogas generation [1,2]. Biogas is a mixture of different gases, mainly methane and carbon dioxide. Its production during anaerobic digestion involves microorganisms, which results in some serious drawbacks of this technology, as the bacteria responsible for methane generation are very sensitive to the environmental conditions, such as oxygen content, pH or presence of certain organic and inorganic compounds [3]. Therefore, wastes or wastewaters containing substances which are toxic or recalcitrant to these microorganisms cannot be used in the traditional biogas production process.

Application of the photocatalytic process instead of the biological one could remove that restriction. Photocatalysis is not selective for any kind of substrates, therefore it might be used for treatment of all contaminants, even those which are toxic to the methanogenic bacteria [4].

Due to its significant activity, stability and low cost TiO_2_ is widely used as a photocatalyst. Most investigations concerning the photocatalytic treatment of organic compounds in aqueous solutions are focused on their complete mineralization to CO_2_ and H_2_O. Usually, during these experiments the composition of the aqueous phase is only monitored. However, determination of the gas phase composition should be also of interest. There are some reports [5,6,7,8,9,10,11] showing that the process of a photocatalytic reduction of CO_2_ may lead to methane formation.

The first papers concerning the photocatalytic generation of hydrocarbons from organics in liquid phase were published in the 1970s by Kraeutler and Bard [12,13,14]. These authors described a photocatalytic decarboxylation of acetic acid under UV light in the presence of Pt/TiO_2_ photocatalyst. The reaction in which CH_4_ and CO_2_ were evolved as the products was named the “photo-Kolbe” reaction. A few years later Sakata *et al.* [15] reported methane and ethane formation during photodecomposition of acetic and propionic acids in the presence of bare and Pt modified TiO_2_.

A possibility of hydrocarbon formation during photodegradation of C_1_–C_3_ alcohols in aqueous suspensions of TiO_2_ was investigated by Dey and Pushpa [16]. They concluded that CH_4_ and CO_2_ were the main products of the reaction of methanol, ethanol and 2-propanol. Other hydrocarbons such as ethane, ethene and propene were also detected; however, at relatively low yields. Similar investigations were conducted by Bahruji *et al.* [17]. The authors used Pt–modified TiO_2_ in order to increase H_2_ formation. CH_4_, CO_2_, C_2_H_6_ and C_3_H_8_ were also identified in the gas phase.

Xu *et al.* [18] reported biomass reforming on Pt/TiO_2_ (anatase-rutile structure) leading to H_2_ generation. Methanol, propanetriol, formic acid and glucose were used as the model compounds and sacrificial agents. The possibility of hydrogen production from glucose, sucrose and starch over noble metal-loaded TiO_2_ photocatalysts was also described by Fu *et al.* [19]. The results revealed an enhancement of H_2_ production in case of Pd and Pt modified TiO_2_ and an inhibition of the efficiency in aerated systems.

Recently, Klauson *et al.* [20] described the application of TiO_2_ modified with Pt, Co, W, Cu or Fe for the production of hydrogen, oxygen and low molecular weight hydrocarbons from aqueous solutions of humic substances under anoxic conditions. In the presence of all the above materials the formation of CH_4_ was observed, although the highest yield was found in case of Pt-TiO_2_. That photocatalyst was also the most efficient when formation of C_2_H_4_, C_2_H_6_ and H_2_ was taken into account.

In the present work an Fe-modified TiO_2_ photocatalyst was applied for the photocatalytic generation of useful hydrocarbons and hydrogen which could be regarded as the potential source of “green energy”. Different organics representing biomass-derived compounds, including an aliphatic acid (acetic acid), aliphatic alcohols (methanol and ethanol) and glucose were used in the experiments. In particular the influence of the reaction atmosphere on the products evolution was investigated. The Fe/TiO_2_ photocatalyst was chosen on a basis of our previous investigations [21] during which we found that it exhibits high activity in the “photo-Kolbe” reaction using acetic acid as a substrate.

## 2. Results and Discussion

### 2.1. Photocatalytic Decomposition of Various Organic Compounds: The Influence of a Substrate on the Formation of the Gaseous and Liquid Products

Depending on the substrate, different gases were identified in the headspace volume of the reactor (Table 1). In case of acetic acid, the main products of its decomposition were CH_4_ and CO_2_. Low amounts of C_2_H_6_, C_3_H_8_ and H_2_ were also identified. During the photocatalytic degradation of alcohols the following gaseous products were identified: CO_2_, CH_4_ and H_2_ in case of CH_3_OH and CO_2_, CH_4_, C_2_H_6_ and H_2_ in case of C_2_H_5_OH. The gaseous products formed during photodegradation of C_6_H_12_O_6_ were CH_4_, CO_2_ and H_2_ (Table 1). The diversity of the products generated from the applied substrates resulted from their different photocatalytic decomposition pathways.

**Table 1:** Products identified in the gas and liquid phases after 27 h of irradiation over Fe/TiO_2_.

Substrate	Composition of a Gas Phase	Composition of a Liquid Phase
CH_3_COOH	CH_4_, CO_2_, C_2_H_6_, C_3_H_8_, H_2_	CH_3_COOH, CH_3_OH, C_2_H_5_OH, CO(CH_3_)_2_, CH_3_CHO, CH_3_COOCH_3_
CH_3_OH	CH_4_^a^, CO_2_, H_2_	CH_3_OH, CH_3_CHO
C_2_H_5_OH	CH_4_, CO_2_, C_2_H_6_, H_2_	C_2_H_5_OH, CH_3_CHO, CH_3_OH
C_6_H_12_O_6_	CH_4_, CO_2_, H_2_	C_6_H_12_O_6_, CH_3_CHO, C_2_H_5_OH, CH_3_COOCH_3_

^a^ in air atmosphere only.

Taking into consideration that some by-products of the organics’ degradation must have been generated in the liquid phase, the composition of the reaction solution was also examined. The investigations revealed (Table 1) the presence of trace amounts of acetaldehyde (CH_3_CHO) in all cases. Furthermore, methanol (CH_3_OH) in the case of acetic acid and ethanol decomposition, and ethanol (C_2_H_5_OH) and methyl acetate (CH_3_COOCH_3_) in the case of acetic acid and glucose degradation were identified. In addition, small quantities of acetone (CO(CH_3_)_2_) were detected during the photodecomposition of acetic acid. The amounts of all the products in the liquid phase were very low and no clear dependence of the liquid phase composition on the reaction atmosphere used was found.

### 2.2. Effect of the Reaction Atmosphere on Gas Phase Composition during the Photodegradation of Various Organic Substrates

**Figure 1:**
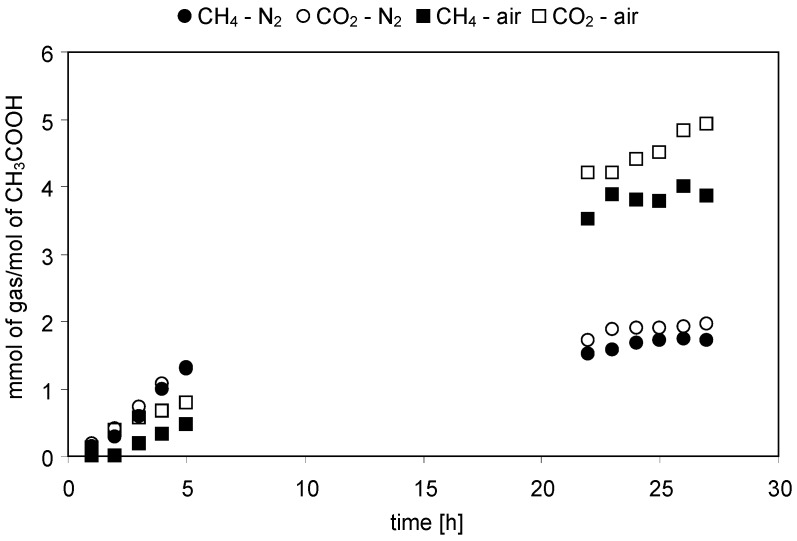
Evolution of CH_4_ and CO_2_ in time of irradiation during the photocatalytic degradation of CH_3_COOH. Photocatalyst loading: 1g/dm^3^; CH_3_COOH concentration: 1 mol/dm^3^; solution pH: 2.6; t = 25 °C.

**Figure 2:**
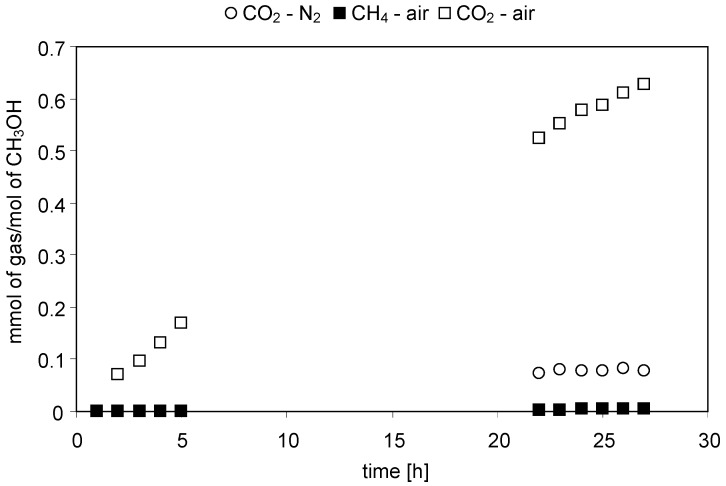
Evolution of CH_4_ and CO_2_ in time of irradiation during the photocatalytic degradation of CH_3_OH. Photocatalyst loading: 1g/dm^3^; CH_3_OH concentration: 1 mol/dm^3^; solution pH: 6.3; t = 25 °C.

**Figure 3:**
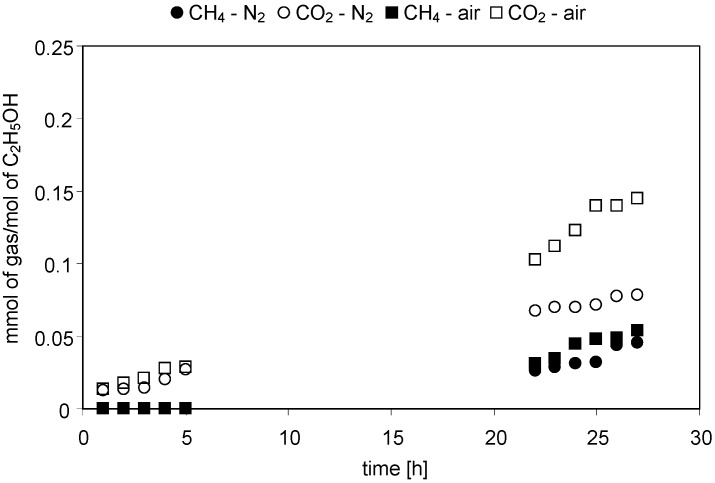
Evolution of CH_4_ and CO_2_ in time of irradiation during the photocatalytic degradation of C_2_H_5_OH. Photocatalyst loading: 1g/dm^3^; C_2_H_5_OH concentration: 1 mol/dm^3^; solution pH: 4.8; t = 25 °C.

**Figure 4:**
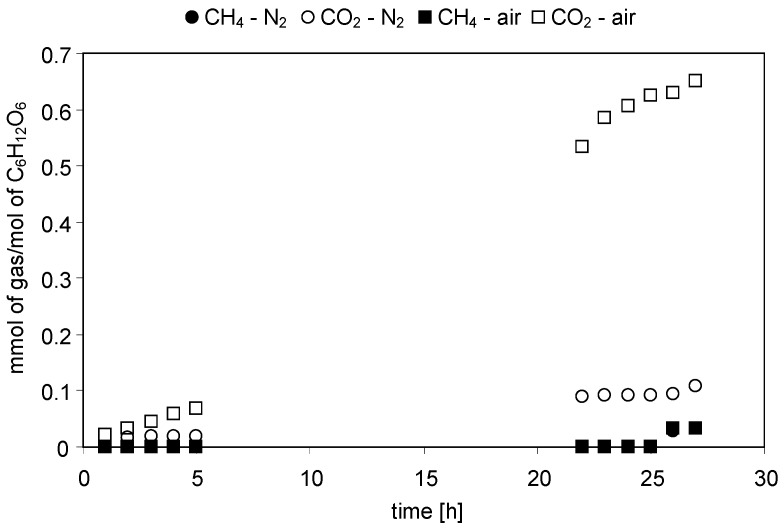
Evolution of CH_4_ and CO_2_ in time of irradiation during the photocatalytic degradation of C_6_H_12_O_6_. Photocatalyst loading: 1 g/dm^3^; C_6_H_12_O_6_ concentration: 1 mol/dm^3^; solution pH: 5.4; t = 25 °C.

**Figure 5:**
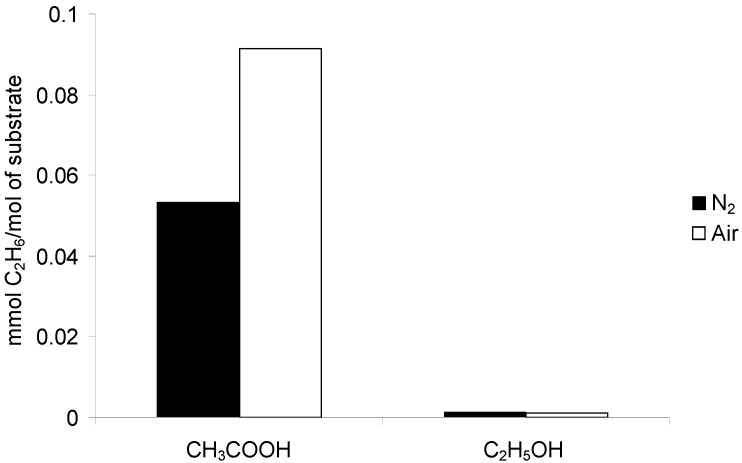
Comparison of the amounts of C_2_H_6_ evolved during the photocatalytic degradation of various organic substrates after 27 h of irradiation in the presence of Fe/TiO_2_. Photocatalyst loading: 1g/dm^3^; substrate concentration: 1 mol/dm^3^; t = 25 °C.

**Figure 6:**
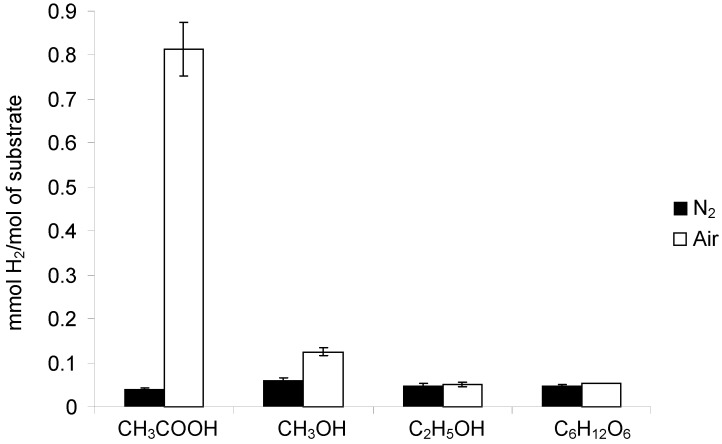
Comparison of the amounts of H_2_ evolved during the photocatalytic degradation of various organic substrates after 27 h of irradiation in the presence of Fe/TiO_2_. Photocatalyst loading: 1g/dm^3^; substrate concentration: 1 mol/dm^3^; t = 25 °C.

The concentrations of gaseous reaction products evolved with time of irradiation were continuously monitored during the experiments. Figure 1, Figure 2, Figure 3 and Figure 4 present changes of the amounts of CO_2_ and CH_4_ in the gaseous phase during the processes conducted under either N_2_ or air atmospheres. In Figure 5 and Figure 6 a comparison of the amounts of C _2_H_6_ and H_2_ evolved after 27 h of the decomposition of the model compounds is shown.

#### 2.2.1. Acetic Acid

In general, the main mechanism responsible for a photocatalytic decomposition of CH_3_COOH is its decarboxylation initiated by the photogenerated holes (h^+^). This reaction, known as the “photo–Kolbe” reaction, leads to the production of one mole of CO_2_ and one mole of CH_4_ from one mole of CH_3_COOH:

(1) $$CH_{3}COOH\to CH_{4}+CO_{2}$$

Moreover, recombination of methyl radicals might take place, which results in a formation of C_2_H_6_, except from CH_4_ [12,13,14,15,21,22,23,24]. Formation of C_2_H_6_ and H_2_ can be written as follows:

(2) $$2CH_{3}COOH\to C_{2}H_{6}+2CO_{2}+H_{2}$$

Further, as can be seen in Table 1, formation of C_3_H_8_ can also occur. A possible mechanism of propane generation can be as follows [15]:

(3) $$C{H_{3}}^{•}+CH_{3}COOH\to CH_{4}{+}^{•}CH_{2}COOH$$

or:

(4) $$OH^{•}+CH_{3}COOH\to H_{2}O{+}^{•}CH_{2}COOH$$

(5) $${}^{•}CH_{2}COOH+C{H_{3}}^{•}\to C_{2}H_{5}COOH$$

(6) $$C_{2}H_{5}COOH+h^{+}\to^{•}C_{2}H_{5}+CO_{2}+H^{+}$$

(7) $${}^{•}C_{2}H_{5}+C{H_{3}}^{•}\to C_{3}H_{8}$$

Nevertheless, the present results clearly show that the CH_4_/CO_2_ ratio after 27 h of irradiation was 0.88 when a N_2_ atmosphere was applied and 0.78 when the process was conducted in the presence of air. This suggests that reaction (1) was not the only one proceeding in the system. From Table 1 it can be found that aside from methane, ethane was also formed. In this process methyl radicals are consumed. Therefore, the amount of ethane should be also taken into consideration. Assuming that two methyl radicals form one molecule of C_2_H_6_ the CH_3_^•^/CO_2_ ratio can be calculated. After 27 h of irradiation of acetic acid solution the amount of C_2_H_6_ evolved in a N_2_ atmosphere was 0.05 mmol C_2_H_6_/mol CH_3_COOH, whereas under aerated conditions it was 0.09 mmol C_2_H_6_/mol CH_3_COOH. Thus, the CH_3_^•^/CO_2_ ratio was 0.93 and 0.82 for N_2_ and air atmosphere, respectively. However, the values are still below 1. Incorporation of C_3_H_8_ in the calculations also does not allow one to get a ratio of 1, since the amount of propane was an order of magnitude lower than that of ethane. These results suggest that formation of carbon dioxide might also be due to the mineralization of CH_3_COOH to H_2_O and CO_2_:

(8) $$CH_{3}COOH+2O_{2}\to 2CO_{2}+2H_{2}O$$

Reaction (8) is understandable when the aerated conditions are considered; however, the obtained results revealed that it also proceeded in the N_2_-purged system. In our previous paper [21] we have discussed higher evolution rate of CO_2_ compared to CH_4_ by the reaction of CH_3_COOH with the photogenerated oxygen. This O_2_ as well as the hydroxyl radicals might be responsible for the mineralization of CH_3_COOH [21], which leads to higher CO_2_ evolution.

The results shown in Figure 1 revealed that the amounts of CH_4_ and CO_2_ evolved under aerated conditions were more than two times higher compared to a N_2_ atmosphere (1.72 *vs.* 3.85 mmolCH_4_/molCH_3_COOH and 1.95 *vs.* 4.93 mmolCO_2_/molCH_3_COOH, respectively, after 27 h). Higher efficiency of CH_4_ evolution under the aerated conditions can be explained by more effective separation of e^−^/h^+^ pairs in the presence of O_2_, being an efficient electron scavenger, and acetic acid, which is known as an effective hole scavenger. Therefore, in the presence of both oxygen and CH_3_COOH the “photo–Kolbe reaction” should occur more easily, what was confirmed by the results presented in Figure 1. Moreover, it was found that the concentration of O_2_ in the headspace volume of the reactor decreased from 21 to 12 vol.% after 27 h of irradiation, which confirms that oxygen was consumed in the process.

The obtained results (Figure 5) also revealed higher efficiency of C_2_H_6_ evolution in the aerated compared to the N_2_ purged system. Ethane formation (Reaction (2)) is initiated by the photogenerated holes, therefore, can easily proceed under both deaerated and aerated conditions. However, like in case of methane, more efficient separation of e^−^/h^+^ pairs contributes to the enhancement of ethane formation. Moreover, the presence of O_2_ can result in the increase of the amount of C_2_H_6_ by enabling of its formation according to the following equation [23,24]:

(9) $$2CH_{3}COOH+\frac{1}{2}O_{2}\to C_{2}H_{6}+2CO_{2}+H_{2}O$$

As shown in Table 1, amongst the products of CH_3_COOH decomposition hydrogen was also present. As in case of other gases, evolution of H_2_ was significantly higher in an air atmosphere compared to a N_2_ one (Figure 6). After 27 h of irradiation the amounts of H_2_ were 0.04 and 0.81 mmolH_2_/mol CH_3_COOH in N_2_ and air purged system, respectively. The data discussed above show that the photocatalytic conversion of CH_3_COOH into hydrocarbons and hydrogen was significantly more effective in the presence of air than in the N_2_ purged system.

#### 2.2.2. Methanol

The photocatalytic degradation of methanol under deaerated conditions can be written as [25]:

(10) $$CH_{3}OH+H_{2}O\to CO_{2}+3H_{2}$$

This reaction can also be represented as two half-reactions of oxidation and reduction, respectively:

(11) $$CH_{3}OH+H_{2}O+6h^{+}\to CO_{2}+6H^{+}$$

(12) $$6H^{+}+6e^{-}\to 3H_{2}$$

As reported by Chen *et al.* [25], Reaction (12) cannot occur easily in an aerated system because only few hydrogen atoms are formed in the presence of oxygen. Under such conditions, oxygen is more competitive in capturing the photogenerated electrons, which eventually leads to the formation of H_2_O_2_ and OH^•^.

The obtained results (Table 1) revealed formation of CO_2_ and H_2_ as the only gaseous products of CH_3_OH decomposition in N_2_ atmosphere, which confirms the mechanism presented by Equations (10)–(12). Nonetheless, if the only reaction occurring in the investigated system were Reaction (10), the H_2_/CO_2_ ratio should be equal to 3, but the experimental data show that the ratio is significantly lower (*ca.* 0.7–0.8). This suggests that some other reactions proceeded in the system. As in case of CH_3_COOH, such a reaction can be mineralization of CH_3_OH yielding CO_2_ and H_2_O as products [24]:

(13) $$CH_{3}OH+1\frac{1}{2}O_{2}\to CO_{2}+2H_{2}O$$

During the experiments conducted in the air-purged system, the evolution of methane, except from CO_2_ and H_2_, was observed (Figure 2). Its concentration in the gaseous mixture was, however, very low and after 27 h of irradiation it only amounted to 4.26 μmol/molCH_3_OH. Nonetheless, the observed formation of CH_4_ might lead to a conclusion that the mechanism of methanol decomposition in the presence of air is not as simple as the one described by Equation (13). For example, a possibility of CO_2_ photoreduction cannot be excluded here [16]. Dey and Pushpa reported that carbon dioxide, generated during mineralization of methanol, could undergo a methanation reaction by e^−^ and yield CH_4_. In the case of greater amounts of CO_2_ (as is the case in this work, when the system was aerated) there is a better chance of it being reduced, which can explain the results shown in Figure 2.

The amount of CO_2_ evolved in the presence of air was at the end of the experiment about eight times higher compared to the N_2_ atmosphere (0.627 *vs.* 0.078 mmolCO_2_/mol CH_3_OH, respectively). High CO_2_ evolution was an effect of methanol mineralization (Equation (13)) and was accompanied by a decrease of O_2_ concentration in the gaseous phase (from 21 to 16 vol.%). It was also observed that in the presence of N_2_ no gaseous product evolved from the reaction mixture within the initial 5 h of the experiment. On the contrary, when the reaction was conducted under aerated conditions the evolution of CO_2_ started after 2 h of irradiation.

Evolution of hydrogen was significantly lower compared to that of CO_2_ (Figure 6). After 27 h of irradiation the amount of H_2_ was 0.06 and 0.13 mmolH_2_/mol CH_3_OH in the N_2_ and air purged systems, respectively.

#### 2.2.3. Ethanol

In the case of ethanol, the main products identified in the gaseous mixture were CH_4_ and CO_2_ (Figure 3). Moreover, some amounts of C_2_H_6_ and H_2_ were also identified (Figure 5 and Figure 6). The CH_4_/CO_2_ ratio was higher in N_2_ than in an air atmosphere and amounted to 0.83 and 0.05, respectively. This resulted from significantly higher CO_2_ evolution in the presence of air compared to the N_2_-purged system (1.45 *vs.* 0.078 mmolCO_2_/mol C_2_H_5_OH after 27 h). As in case of other substrates a decrease of O_2_ concentration in the gaseous phase in case of the experiments conducted under aerated conditions was found (from 21 to 15 vol.%). It was also observed that the amount of methane obtained under both conditions was comparable (Figure 3).

Decomposition of ethanol is more complex compared to methanol due to the presence of the ethyl group in the C_2_H_5_OH structure. As a result, the range of intermediate degradation products is very wide [25]. In case of the deaerated conditions the overall reaction of ethanol decomposition can be written as follows [17,24]:

(14) $$C_{2}H_{5}OH+H_{2}O\to CO_{2}+2H_{2}+CH_{4}$$

The reduction reaction can be represented by Equation (12), like in case of methanol [25]. However, the oxidation reactions are different. Generally, methane can be produced either by the reaction of free methyl radicals with H^•^ or ethanol, or the reaction of acetic radicals with ethanol [24]. In case of the present research, since acetic acid was not identified in the liquid phase (Table 1), the most probable pathway of CH_4_ formation was the one involving CH_3_^•^ and C_2_H_5_OH. Moreover, as in case of methanol [16], the reduction of CO_2_ leading to the methane production cannot be ignored here. Furthermore, methyl radicals can also recombine yielding C_2_H_6_ as the product (Figure 5).

In the presence of oxygen the decomposition of ethanol can be described by the following equation [24]:

(15) $$2C_{2}H_{5}OH+1\frac{1}{2}O_{2}\to CH_{4}+CO_{2}+2H_{2}O+CH_{3}CHO$$

Equation (14) indicates that H_2_ should be present amongst the ethanol decomposition products. Indeed, the analysis of the gaseous phase composition revealed evolution of hydrogen under both the aerated and deaerated conditions (Figure 6). Furthermore, H_2_ could be produced by a degradation of the intermediate products present in the liquid phase (CH_3_CHO, CH_3_OH, Table 1). However, taking into account that their concentrations were very low, this pathway was of minor importance. The amount of H_2_ formed in the N_2_ purged system was comparable to that in the aerated system (0.049 *vs.* 0.051 mmolH_2_/molC_2_H_5_OH, respectively). If we recall the methane evolution under aerated and deaerated conditions (Figure 3) we may find that the reaction atmosphere did not clearly influence the effectiveness of H_2_ and CH_4_ formation during ethanol decomposition.

#### 2.2.4. Glucose

The photocatalytic reforming of C_6_H_12_O_6_ is a very complex process which proceeds through numerous steps, in which intermediates such as carboxylic acids, aldehydes and hydrocarbons are formed [19,26]. A detailed probable mechanism of glucose degradation under anaerobic conditions leading to the formation of H_2_ and CO_2_ was recently discussed by Fu *et al.* [19]. The overall reaction can be written as:

(16) $$C_{6}H_{12}O_{6}+6H_{2}O\to 6CO_{2}+12H_{2}$$

The present research confirmed the formation of CO_2_ and H_2_ (Figure 4 and Figure 6). In addition, small amounts of CH_4_ were identified as well. From Figure 4 it can be found that the amount of CO_2_ was higher in the presence of air compared to a N_2_ atmosphere, which is consistent with the results observed for other substrates. After 27 h of irradiation the amount of CO_2_ in the gaseous mixture was 0.65 mmolCO_2_/molC_6_H_12_O_6_ and 0.11 mmolCO_2_/molC_6_H_12_O_6_ for air and N_2_, respectively. In the experiment conducted under aerated conditions the concentration of O_2_ in the gaseous phase decreased from 21 to 15 vol.% which confirms its consumption during glucose decomposition.

No significant difference between the efficiency of hydrogen evolution in the two systems was observed. In case of the N_2_-purged system the amount of H_2_ was 0.048 mmolH_2_/molC_6_H_12_O_6_, whereas in case of the aerated system, it was 0.054 mmolH_2_/molC_6_H_12_O_6_ (Figure 6). Similarly, no difference in the amount of methane evolved in the presence and in the absence of oxygen was found. After 27 h of the reaction in both N_2_ and air atmospheres, the amount of CH_4_ reached 0.033 mmolCH_4_/molC_6_H_12_O_6_ (Figure 4). The observed evolution of methane can be explained by decomposition of by-products formed in the liquid phase (Table 1) as well as CO_2_ photoreduction, as discussed earlier.

### 2.3. Hydrogen Evolution in the Presence of Oxygen: a Point of Discussion

The results discussed above revealed that the presence of oxygen at a concentration of 21 vol.% or less (*i.e.*, oxygen in air) did not suppress hydrogen evolution during the photodegradation of organic compounds in the performed experiments. What is more, in the cases of acetic acid and methanol a significant enhancement of H_2_ formation was even observed (Figure 6). This is somewhat unusual in view of the electron acceptability of O_2_ and the competitiveness with H^+^ for electron scavenging [16,19,24,25,27].

In order to investigate if the observed phenomenon resulted from the presence of Fe in the photocatalyst structure, an additional experiment was performed. A TiO_2_ photocatalyst prepared in a similar way to the Fe/TiO_2_, but without impregnation with Fe(NO_3_)_3_, was applied in a process of photocatalytic CH_3_COOH degradation under N_2_ and air atmosphere. After 27 h of irradiation it was found that the effectiveness of evolution of CH_4_ and H_2_ in the air purged system was higher by 65 and 45%, respectively, compared to the N_2_ atmosphere. Therefore, it was concluded that the addition of iron was not responsible for the phenomenon described above.

There are very few papers reporting that O_2_ does not affect negatively or could have a positive influence on hydrogen photogeneration [28,29,30]. Korzhak *et al.* [28] found that when a small amount of air was introduced to a photocatalytic system containing ethanol, the yield of H_2_ formation increased. However, in case of mixtures saturated with oxygen or air, hydrogen formation was almost completely suppressed. The authors contributed the observed increase in hydrogen production to the fact that under such conditions the reactions of O_2_ with active free organic radicals take place with high rate constants. Therefore oxygen is consumed mainly in the process leading to the evolution of additional amounts of hydrogen. Moreover, dissolved oxygen might be involved in stabilization of the radical intermediates thus could enhance the reaction efficiency [31]. Furthermore, organic substrates such as acids, alcohols or glucose, contribute to the improvement of charge separation by scavenging of photogenerated holes and consuming O_2_ in diverse direct oxidation reactions, which leads to a decrease of the oxygen concentration [32,33,34].

Anyhow, the majority of the work on hydrogen generation with semiconductors dispersed in a solution is carried out in an oxygen–free atmosphere to avoid the back recombination processes, oxygen interferences with the photocatalyst which occurs while forming of superoxides and/or peroxides and the competition of O_2_ and H^+^ for the reduction sites [15,17,18,19,26,32,35,36,37]. Most of the papers which describe the photocatalytic degradation of organics in the presence of O_2_ are focused on its total mineralization, thus the evolution of H_2_ is not discussed. We have proved that the negative O_2_ influence on the H_2_ generation from different organic substrates is not so evident. In some cases (e.g., decomposition of acetic acid) an increase in H_2_ evolution yield can even be obtained. Therefore, a broad and detailed discussion is needed in order to explain the discussed phenomenon.

## 3. Experimental Section

### 3.1. Photocatalyst

The photocatalyst used in this study was described in details in our previous paper [21]. In brief, the Fe/TiO_2_ was prepared by an impregnation method using crude TiO_2_ obtained from the Chemical Factory “Police” (Police, Poland) and (Fe(NO_3_)_3_) as the Fe precursor. The sample was calcined at 500 °C. The amount of Fe introduced to the sample was 20 wt.%. The Fe/TiO_2_ contained anatase, rutile and Fe_2_O_3_ phases. The crystallite size of anatase and the anatase over rutile ratio were equal to 9 nm and 87:13, respectively. The specific surface area S_BET_ was 82 m^2^/g.

### 3.2. Photocatalytic Reaction

The photocatalytic reaction was conducted in a cylindrical quartz reactor (type UV-RS-2, Heraeus, Hanau, Germany) equipped with a medium pressure mercury vapour lamp (TQ-150, λ_max_ = 365 nm). The total volume of the reactor was 765 cm^3^ (350 cm^3^ of a liquid phase and 415 cm^3^ of headspace). In the upper part of the reactor a gas sampling port was mounted. At the beginning of the experiment 0.35 dm^3^ of CH_3_COOH, CH_3_OH, C_2_H_5_OH or C_6_H_12_O_6_ solution and 1 g/dm^3^ of the photocatalyst were introduced into the reactor. The concentration of the organic substrates was 1 mol/dm^3^ in all the experiments.

Before the photocatalytic reaction N_2_ (in order to eliminate the dissolved oxygen) or air were bubbled through the reactor for 1 h. Then, the gas flow was stopped and UV lamp, positioned in the centre of the reactor, was turned on to start the photoreaction. The process was conducted for 27 h. The reaction mixture containing the photocatalyst in suspension was continuously stirred during the experiment by means of a magnetic stirrer. All the experiments were repeated at least twice in order to confirm the reproducibility of the results. Gaseous products of the reaction were analyzed using a SRI 8610C GC (SRI Instruments, Torrance, CA, USA) equipped with TCD and HID detectors, and Shincarbon (carbon molecular sieve; 2 m, 1 mm, 100–120 mesh), molecular sieve 5 Å (3 m, 2 mm, 80–100 mesh) and 13× (1.8 m, 2 mm, 80–100 mesh) columns. Helium was used as the carrier gas. The composition of the liquid phase was determined using a SRI 8610C GC equipped with a FID detector and a MXT^®^-1301 (60 m) column. Hydrogen was used as the carrier gas.

## 4. Conclusions

The possibility of photocatalytic generation of combustible hydrocarbons and hydrogen from various organic substrates, including an aliphatic acid (CH_3_COOH), alcohols (CH_3_OH, C_2_H_5_OH) and sugar (C_6_H_12_O_6_) was demonstrated. The composition of the gaseous phase was influenced by both the applied substrate and the reaction atmosphere. In general, higher efficiency of hydrocarbon and hydrogen generation was obtained under aerated conditions, which is very advantageous from the point of view of possible future applications. In the presence of air, the gaseous phase contained CO_2_, CH_4_ and H_2_, regardless of the substrate used. Moreover, formation of C_2_H_6_ and C_3_H_8_ in the case of acetic acid and C_2_H_6_ in the case of ethanol was observed.

The obtained results revealed that the presence of oxygen did not suppress hydrogen evolution during the photodegradation of organic compounds. In the cases of acetic acid and methanol a significant enhancement of H_2_ formation was even observed. Further investigations concerning this issue as well as the improvement of the efficiency of the presented system are in progress.
